# Sub-anesthetic dose of esketamine decreases postoperative opioid self-administration after spine surgery: a retrospective cohort analysis

**DOI:** 10.1038/s41598-024-54617-5

**Published:** 2024-02-16

**Authors:** Hongyu Zheng, Peng Zhang, Shengnan Shi, Xue Zhang, Qiang Cai, Xingrui Gong

**Affiliations:** 1 Department of Anesthesiology, Institution of Neuroscience and Brain Disease, Xiangyang Central Hospital, Affiliated Hospital of Hubei University of Arts and Science, Xiangyang, Hubei China; 2 Department of Emergency, Xiangyang Central Hospital, Affiliated Hospital of Hubei University of Arts and Science, Xiangyang, Hubei China; 3 Department of Orthopedics, Xiangyang Central Hospital, Affiliated Hospital of Hubei University of Arts and Science, Xiangyang, China

**Keywords:** Esketamine, Spine surgery, Patient-controlled analgesia, Opioid, Anesthesia, Neuroscience, Diseases, Medical research

## Abstract

The use of intraoperative sub-anesthetic esketamine for postoperative analgesia is controversial. In this study, the impact of sub-anesthetic esketamine on postoperative opioid self-administration was determined. Patients who underwent spinal surgery with patient-controlled analgesia (PCA) from January 2019 to December 2021 were respectively screened for analysis. Postoperative PCA was compared between patients who received a sub-anesthetic esketamine dose and patients who were not treated with esketamine (non-esketamine group) with or without propensity score matching. Negative binomial regression analysis was used to identify factors associated with postoperative PCA. Patients who received intraoperative sub-anesthetic esketamine self-administered less PCA (*P* = 0.001). Azasetron, esketamine, and dexamethasone lowered the self-administration of PCA (IRR with 95% confidential interval, 0.789 [0.624, 0.993]; 0.581 [0.458, 0.741]; and 0.777 [0.627, 0.959], respectively). Fixation surgery and drinking were risk factors for postoperative PCA (1.737 [1.373, 2.188] and 1.332 [1.032, 1.737] for fixation surgery and drinking, respectively). An intraoperative sub-anesthetic dose of esketamine decreases postoperative opioid self-administration. Azasetron and dexamethasone also decrease postoperative opioid consumption. The study is registered at www.chictr.org.cn (ChiCTR2300068733).

## Introduction

Postoperative pain is a common complication after spinal surgery. The postoperative pain significantly hinders postoperative rehabilitation and affects overall physical and mental health^1^. Effective postoperative pain management is challenging for clinicians, and many patients suffer from moderate to severe pain after surgery^2^. Postoperative pain is a complex physiological and psychological response, arising from multiple factors, including wound stimulation, inflammatory response, and increased neural sensitivity. In severe cases, postoperative pain can lead to serious complications, including chronic pain and deep vein thrombosis^3^. Opioids are the most widely used medication for postoperative analgesia. However, complications, including respiration depression, constipation, tolerance, hyperalgesia, and addiction, limit the use of opioids. Non-opioid analgesic adjuvants are a promising approach for postoperative analgesia to avoid opioid-related complications.

Esketamine is used to treat depression and depressive disorders. Recently, esketamine has been used for postoperative analgesia. We previously demonstrated that a sub-anesthetic dose of esketamine decreases pain and anxiety after thyroid and breast surgery^4^. However, a recent large multi-center study demonstrated that intraoperative ketamine did not exhibit any analgesic effects^5^. In a recent meta-analysis, intravenous esketamine decreased pain intensity and opioid requirements after surgery, but the level of certainty was moderate to low^6^. Thus, the effects of intraoperative esketamine on postoperative pain are unclear. A high dose of esketamine may increase postoperative complications. Therefore, we determined the impact of a sub-anesthetic dose of intraoperative esketamine on postoperative opioid self-administration in the setting of patient-controlled analgesia (PCA) after spinal surgery.

## Methods

This study followed the Declaration of Helsinki and was approved by the Ethics Committee of Xiangyang Central Hospital, which is affiliated with Hubei University of Arts and Science. The study was registered at www.chictr.org.cn (ChiCTR2300068733). Patient written informed consent was waived by the Ethics Committee of Xiangyang Central Hospital, and patient information was deidentified before the data analysis.

Patients who underwent spinal surgery with postoperative PCA in our tertiary hospital from January 2019 to December 2021 were eligible for the study. Patient exclusion criteria included extubation failure after surgery. The PCA pump (Jiangsu Renxian Medical Manufacture Technology) recorded information about the time and the number of PCA pushes. All patients were returned to the wards with a numerical rating scale (NRS) for pain of less than 4 and were educated to push the button if their NRS was equal to or higher than 4. The 100-mL PCA pump contained 10 mg of hydromorphone and 20 mg of azasetron. The parameters were set at a rate of 2 mL/h, 1.5 mL per push, and a lock-out time of 15 min.

All patients used propofol, midazolam, sufentanil, rocuronium bromide, cisatracurium, and sevoflurane for anesthesia induction and maintenance. Patient information about intraoperative adjuvants was retrospectively collected from electronic records. The use of anesthesia adjuvants, including azasetron 10 mg, esketamine 0.2–0.5 mg/kg, dexamethasone 10 mg, flurbiprofen 50 mg, dezocine 5 mg, hydromorphone 0.4 mg, and dexmedetomidine 0.5 μg/kg/h, was determined by the anesthetists. Surgery types included internal fixation and non-fixation surgeries. Patient demographics (sex, age, BMI, and weight), previous comorbidities (smoking, drinking, lumbar surgery history, cerebrovascular disease, heart disease, pulmonary disease, hepatic disease, renal disease, hypertension, and diabetes mellitus [DM]), previous psychiatric illness (schizophrenia and depression), patient status (American Society of Anesthesiologists [ASA] physical status, heart function), laboratory test results (white blood cell count [WBC], neutrophil, uric acid, C-reactive protein [CRP]), surgery information (surgery duration, surgery with fixation or not), other intraoperative anesthesia adjuvants (azasetron, dexamethasone, flurbiprofen, dezocine, hydromorphine, and dexmedetomidine), and preoperative and postoperative analgesia usage (nonsteroidal antiinflammatory drug [NSAID] and opioid) were collected from the electronic health database and included in the relevant factor analysis. The follow-up time was 48 h after surgery.

### Statistical analysis

Continuous and categorical data are presented as means (standard deviation, SD) and numbers (interquartile range, IQR), respectively. Propensity score matching was used as a sensitivity analysis. The “MatchIt” package for the R statistical software package was utilized for propensity score matching. Patient demographics, previous comorbidities, patient status, laboratory test results, intraoperative anesthesia adjuvants, and surgery information were adjusted with the nearest neighbor, and a caliper width of 0.2 standard deviations of the logit of the propensity score was used. Univariate and multivariable negative binomial regression analyses (“MASS” package for the R statistical software package) were used to identify factors associated with postoperative PCA use. In addition, PCA use was compared between different esketamine doses. Surgery type and sex-specific effects of esketamine and other therapies were analyzed. A *P* < 0.05 indicated a statistical difference.

### Ethics approval and consent to participate

The study was approved by the ethics committee of Xiangyang Central Hospital, Affiliated with Hubei University of Arts and Science.

## Results

### Baseline characteristics

Patient baseline characteristics are shown in Table 1. During the study period, 590 patients received PCA after spinal surgery, and 121 patients received esketamine. Approximately half of patients (55%) were female. Fewer patients received flurbiprofen and more patients underwent fixation surgery in the esketamine group compared with the non-esketamine groups (*P* < 0.05). No significant differences in demographics (sex, age, BMI, or weight), previous comorbidities (smoking, drinking, lumbar surgery history, cerebrovascular disease, heart disease, pulmonary disease, hepatic disease, renal disease, hypertension, or DM), patient status (ASA physical status or heart function), laboratory test results (WBC, neutrophil, uric acid, or CRP), preoperative NSAID usage, surgery parameters (surgery duration or surgery with fixation), or other intraoperative anesthesia adjuvants (azasetron, dexamethasone, flurbiprofen, dezocine, hydromorphine, or dexmedetomidine) were detected. No patients were diagnosed with preoperative psychiatry illness, including schizophrenia, and depression, and no patients received opioids for analgesia before surgery. Postoperative PCA (the number of PCA pushes) was lower in the esketamine group compared with PCA use in the non-esketamine group (2.00 [0.00, 3.00] vs. 3.00 [1.00, 7.00], *P* = 0.001, Table 1). In addition, the postoperative NSAID requirement was less in the esketamine group compared with the non-esketamine group (19.8 vs. 33.7%, *P* = 0.005).

**Table 1:** Patient baseline characteristics.

	Non-esketamine (n = 469)	Esketamine (n = 121)	P
Sex male/female (%)	209/260 (44.6/55.4)	55/66 (45.5/54.5)	0.942
Age (median [IQR])	60.00 [52.00, 68.00]	59.00 [52.00, 68.00]	0.681
BMI (median [IQR])	24.00 [22.31, 26.04]	24.00 [22.49, 26.17]	0.922
Weight (median [IQR])	65.00 [58.00, 71.00]	65.00 [59.00, 72.00]	0.513
Smoking no/yes (%)	414/55 (88.3/11.7)	108/13 (89.3/10.7)	0.887
Drinking no/yes (%)	394/75 (84.0/16.0)	109/12 (90.1/9.9)	0.124
Lumbar surgery history no/yes (%)	444/25 (94.7/5.3)	112/9 (92.6/7.4)	0.504
Cerebrovascular disease (%)			0.513
No	433 (92.3)	115 (95.0)	
Ischemic disease	29 (6.2)	4 (3.3)	
Cerebral hemorrhage	5 (1.1)	2 (1.7)	
Others	2 (0.4)	0 (0.0)	
Heart disease (%)			0.313
No	431 (91.9)	108 (89.3)	
Coronary artery disease	32 (6.8)	13 (10.7)	
Valvular disease	3 (0.6)	0 (0.0)	
Others	3 (0.6)	0 (0.0)	
Pulmonary disease no/yes (%)	441/28 (94.0/6.0)	115/6 (95.0/5.0)	0.836
Hepatic disease no/yes (%)	461/8 (98.3/1.7)	119/2 (98.3/1.7)	1
Renal disease no/yes (%)	462/7 (98.5/1.5)	116/5 (95.9/4.1)	0.141
Hypertension no/yes (%)	327/142 (69.7/30.3)	83/38 (68.6/31.4)	0.897
DM no/yes (%)	426/43 (90.8/9.2)	105/16 (86.8/13.2)	0.248
ASA (%)			0.643
II	214 (45.6)	61 (50.4)	
III	238 (50.7)	56 (46.3)	
IV	17 (3.6)	4 (3.3)	
Heart function (%)			0.348
I	352 (75.1)	87 (71.9)	
II	112 (23.9)	34 (28.1)	
III	5 (1.1)	0 (0.0)	
WBC (median [IQR])	5.73 [4.72, 7.03]	5.43 [4.41, 6.76]	0.085
Neutrophil (median [IQR])	3.50 [2.65, 4.64]	3.18 [2.43, 4.39]	0.149
Uric acid (median [IQR])	281.40 [233.20, 340.70]	284.70 [214.70, 334.80]	0.677
CRP (median [IQR])	2.13 [1.44, 3.91]	2.03 [1.39, 3.97]	0.578
Preoperative NSAID no/yes (%)	152/317 (32.4/67.6)	39/82 (32.2/67.8)	1
Azasetron no/yes (%)	103/366 (22.0/78.0)	25/96 (20.7/79.3)	0.853
Dexamethasone no/yes (%)	139/330 (29.6/70.4)	29/92 (24.0/76.0)	0.263
Flurbiprofen no/yes (%)	108/361 (23.0/77.0)	41/80 (33.9/66.1)	0.02
Dezocine no/yes (%)	343/126 (73.1/26.9)	89/32 (73.6/26.4)	1
Hydromorphine no/yes (%)	321/148 (68.4/31.6)	72/49 (59.5/40.5)	0.08
Dexmedetomidine no/yes (%)	370/99 (78.9/21.1)	91/30 (75.2/24.8)	0.453
Surgery duration (median [IQR])	216 [177, 260]	220 [190, 256]	0.599
Surgery type no/fixation (%)	123/346 (26.2/73.8)	20/101 (16.5/83.5)	0.036
Postoperative NSAID no/yes (%)	311/158 (66.3/33.7)	97/24 (80.2/19.8)	0.005
Pushes of PCA (median [IQR])	3.00 [1.00, 7.00]	2.00 [0.00, 3.00]	0.001

*ASA* American Society of Anesthesiologists, *DM* diabetes mellitus, *IQR* interquartile range, *PCA* patient-controlled analgesia, *WBC* white blood cell.

### Baseline characteristics adjusted with propensity score matching

Because the data was imbalanced, variables were adjusted using propensity score matching. The baseline characteristics of 120 patient pairs identified by propensity score matching are listed in Table 2. No significant differences in demographics, previous comorbidities, patient status, laboratory test results, surgery information, and other intraoperative anesthesia adjuvants were detected between the two groups. The number of PCA pushes was higher in the esketamine group compared with the number of pushes in the non-esketamine group (2.00 [0.00, 3.00] vs. 3.00 [1.00, 6.25], *P* = 0.001, Table 2). However, postoperative NSAID requirements were not different between the two groups (esketamine, 20.0% vs. non-esketamine, 30.8%, *P* = 0.075).

**Table 2:** Patient baseline characteristics adjusted using propensity score matching.

	Non-esketamine (n = 120)	Esketamine (n = 120)	P
Sex male/female (%)	52/68 (43.3/56.7)	54/66 (45.0/55.0)	0.897
Age (median [IQR])	59.00 [50.00, 66.00]	59.00 [52.00, 68.25]	0.618
BMI (median [IQR])	24.19 [22.04, 26.44]	24.00 [22.49, 26.18]	0.817
Weight (median [IQR])	65.00 [58.00, 73.25]	65.00 [59.75, 72.00]	0.875
Smoking no/yes (%)	111/9 (92.5/7.5)	108/12 (90.0/10.0)	0.648
Drinking no/yes (%)	113/7 (94.2/5.8)	108/12 (90.0/10.0)	0.339
Lumbar surgery history no/yes (%)	112/8 (93.3/6.7)	111/9 (92.5/7.5)	1
Cerebrovascular disease (%)			0.375
No	114 (95.0)	114 (95.0)	
Ischemic disease	5 (4.2)	4 (3.3)	
Cerebral hemorrhage	0 (0.0)	2 (1.7)	
	1 (0.8)	0 (0.0)	
Heart disease no/coronary artery disease (%)			0.595
No	107 (89.2)	107 (89.2)	
Coronary artery disease	12 (10.0)	13 (10.8)	
Valvular disease	1 (0.8)	0 (0.0)	
Pulmonary disease no/yes (%)	115/5 (95.8/4.2)	114/6 (95.0/5.0)	1
Hepatic disease no/yes (%)	117/3 (97.5/2.5)	118/2 (98.3/1.7)	1
Renal disease no/yes (%)	119/1 (99.2/0.8)	115/5 (95.8/4.2)	0.215
Hypertension no/yes (%)	81/39 (67.5/32.5)	82/38 (68.3/31.7)	1
DM no/yes (%)	103/17 (85.8/14.2)	104/16 (86.7/13.3)	1
ASA (%)			0.637
II	61 (50.8)	61 (50.8)	
III	52 (43.3)	55 (45.8)	
IV	7 (5.8)	4 (3.3)	
Heart function (%)			0.341
I	82 (68.3)	86 (71.7)	
II	36 (30.0)	34 (28.3)	
III	2 (1.7)	0 (0.0)	
WBC (median [IQR])	5.27 [4.33, 6.27]	5.48 [4.42, 6.77]	0.445
Neutrophil (median [IQR])	3.24 [2.42, 4.27]	3.24 [2.46, 4.39]	0.669
Uric acid (median [IQR])	287.20 [225.57, 337.40]	283.45 [214.67, 337.80]	0.795
CRP (median [IQR])	1.97 [1.37, 3.13]	2.04 [1.40, 3.97]	0.484
Preoperative NSAID no/yes (%)	34/86 (28.3/71.7)	38/82 (31.7/68.3)	0.673
Azasetron no/yes (%)	24/96 (20.0/80.0)	25/95 (20.8/79.2)	1
Dexamethasone no/yes (%)	28/92 (23.3/76.7)	29/91 (24.2/75.8)	1
Flurbiprofen no/yes (%)	41/79 (34.2/65.8)	40/80 (33.3/66.7)	1
Dezocine no/yes (%)	82/38 (68.3/31.7)	88/32 (73.3/26.7)	0.478
Hydromorphone no/yes (%)	71/49 (59.2/40.8)	71/49 (59.2/40.8)	1
Dexmedetomidine no/yes (%)	88/32 (73.3/26.7)	91/29 (75.8/24.2)	0.767
Surgery duration (median [IQR])	220.00 [172.25, 250.50]	220.00 [188.75, 251.50]	0.539
Surgery type no/fixation (%)	20/100 (16.7/83.3)	20/100 (16.7/83.3)	1
Postoperative NSAID no/yes (%)	83/37 (69.2/30.8)	96/24 (80.0/20.0)	0.075
Pushes of PCA (median [IQR])	3.00 [1.00, 6.25]	2.00 [0.00, 3.00]	0.001

*ASA* American Society of Anesthesiologists, *DM* diabetes mellitus, *IQR* interquartile range, *PCA* patient-controlled analgesia, *WBC* white blood cell.

### Negative binomial regression analysis

The number of PCA pushes exhibited a negative binomial distribution. Therefore, the factors associated with postoperative PCA pushes were identified using negative binomial regression. Univariate negative binomial regression indicated that drinking, heart disease, preoperative NSAID use, azasetron, esketamine, dexamethasone, and fixation surgery were associated with postoperative PCA pushes. Multivariate negative binomial regression showed that azasetron, esketamine, and dexamethasone prevented postoperative PCA pushes, while fixation surgery and drinking were risk factors for postoperative PCA pushes (Table 3). The IRRs (95% confidential interval) were 0.789 [0.624, 0.993] for azasetron, 0.581 [0.458, 0.741] for esketamine, 0.777 [0.627, 0.959] for dexamethasone, 1.737 [1.373, 2.188] for fixation surgery, and 1.332 [1.032, 1.737] for drinking.

**Table 3:** Univariate and multivariate negative binomial regression for factors associated with postoperative PCA pushes.

	Univariate analysis		Multivariate analysis		
	Z	P	IRR (95% CI)	Z	P
Sex	− 1.307	0.191			
Age	− 1.016	0.309			
BMI	− 1.024	0.306			
Weight	− 0.285	0.776			
Smoking	1.059	0.29			
Drinking	2.408	0.016	1.332 (1.032, 1.737)	2.177	0.029
Lumbar surgery history	− 0.41	0.682			
Cerebrovascular disease	− 1.663	0.0963			
Heart disease	− 2.005	0.045	0.871 (0.667, 1.167)	− 1.024	0.306
Pulmonary disease	− 0.074	0.941			
Hepatic disease	0.11	0.913			
Renal disease	1.2	0.23			
Hypertension	0.089	0.929			
DM	− 0.623	0.533			
ASA	− 0.375	0.708			
Heart function	− 0.878	0.38			
WBC	− 0.119	0.905			
Neutrophil	0.143	0.886			
Uric acid	− 0.274	0.784			
CRP	− 0.377	0.707			
Preoperative NSAID	− 2.66	0.008	0.856 (0.700, 1.044)	− 1.538	0.124
Azasetron	− 3.237	0.001	0.789 (0.624, 0.993)	− 1.975	0.048
Esketamine	− 4.589	4.46E−06	0.581 (0.458, 0.741)	− 4.440	9.00E−06
Dexamethasone	− 3.613	0.001	0.777 (0.627, 0.959)	− 2.311	0.021
Flurbiprofen	− 1.526	0.127			
Dezocine	− 0.837	0.403			
Hydromorphone	− 0.484	0.628			
Dexmedetomidine	− 1.78	0.0751			
Surgery duration	0.658	0.511			
Surgery type	5.668	1.45E−08	1.737 (1.373, 2.188)	4.795	1.63E−06

*ASA* American Society of Anesthesiologists, *CI* confidence interval, *DM* diabetes mellitus, *IRRs* incidence rate ratios, *WBC* white blood cell.

### Esketamine dose for postoperative PCA

The effects of different esketamine doses on postoperative PCA were explored. Patients who received higher doses of esketamine used fewer PCA pushes (2.50 [1.00, 5.00] for 0.2 mg/kg, 2.00 [0.50, 3.00] for 0.3 mg/kg, 1.00 [0.00, 2.50] for 0.4 mg/kg, 1.00 [0.00, 3.00] for 0.5 mg/kg, *P* overall = 0.035, *P* for trend = 0.013, Supplementary Table 1).

### Effects of esketamine and other therapies on PCA in different sexes and surgery types

Esketamine (*P* = 0.001 for males, *P* = 0.036 for females, Supplementary Table 2) and dexamethasone (*P* = 0.038 for males, *P* = 0.001 for females) decreased the number of postoperative PCA pushes in both males and females. Azasetron decreased postoperative pushes of PCA in females (2.00 [1.00, 4.00] vs. 4.00 [2.00, 8.75], *P* = 0.001), but not in males (2.00 [1.00, 6.00] vs. 4.50 [1.00, 8.75], *P* = 0.063). No significant differences in the number of PCA pushes related to intraoperative flurbiprofen, dezocine, hydromorphone, and dexmedetomidine were detected between males and females (*P* > 0.05).

Esketamine (*P* = 0.321 for fixation, *P* = 0.001 for non-fixation), azasetron (*P* = 0.141 for fixation, *P* = 0.001 for non-fixation), and dexamethasone (*P* = 0.336 for fixation, *P* = 0.001 for non-fixation) decreased the number of postoperative PCA pushes in patients who underwent fixation surgery, but not in patients who underwent non-fixation surgery (*P* > 0.05, Supplementary Table 3). No significant effects of intraoperative flurbiprofen, dezocine, hydromorphone, and dexmedetomidine on postoperative PCA pushes were detected in patients who underwent fixation or non-fixation surgeries.

## Discussion

Our results demonstrated that an intraoperative sub-anesthetic dose of esketamine decreases postoperative opioid self-administration. Intraoperative azasetron, esketamine, and dexamethasone also reduce postoperative opioid self-administration, while fixation surgery and drinking are risk factors for increased postoperative opioid self-administration.

In a previous study, a sub-anesthetic dose of ketamine did not affect postoperative pain and opioid consumption in patients aged 60 years or older undergoing major surgery^5^. In contrast, a recent systematic review demonstrated that intraoperative ketamine decreases postoperative pain and opioid consumption^7^. The multi-center study with a large sample size did not agree with the single-center study with a small sample size. Our results indicate that intraoperative esketamine is analgesic and decreases postoperative opioid consumption. The opioid-saving effects may be due to antagonism of NMDA receptors. NMDA receptors are critical for pain signal transduction in the central nervous system, and inhibition of NMDA receptors prevents postoperative pain transmission and central sensitization^8^. We previously showed that intraoperative sub-anesthetic doses of esketamine decreased postoperative pain and anxiety. However, postoperative opioid use was not quantified, and the extent of decreased postoperative opioid consumption was unclear.

A recent study demonstrated that postoperative low-dose ketamine reduced hydromorphone requirements during the first 24 h after lumbar surgery in opioid-tolerant patients^9^. In contrast, our experiment was performed in non-opioid-dependent and tolerant patients. In addition, postoperative PCA was used instead of prescription opioids; thus, patient requirements were represented better. Overall, our results provide evidence that intraoperative esketamine decreases postoperative opioid consumption. We also explored the effects of different doses of esketamine on postoperative opioid consumption. Postoperative opioid consumption was significantly different among the four esketamine dose groups. Higher doses of esketamine resulted in less opioid consumption, suggesting that the analgesic effect of esketamine is dose-dependent from 0.2 to 0.5 mg/kg.

In addition to esketamine, the multivariate analysis demonstrated that azasetron and dexamethasone also lowered postoperative opioid self-administration. Azasetron is a 5HT3 receptor antagonist, and a previous study demonstrated that antagonism of 5HT3 receptors prevents inflammation^10^ and opioid antinociceptive tolerance^11,12^. Another study by our team demonstrated that azasetron reduced postoperative acute pain after thoracic surgery (in submission). In addition, several systematic reviews showed that a single dexamethasone dose decreases postoperative pain and opioid self-administration^13–15^. Dexamethasone may decrease postoperative pain by inhibiting the immune system response to surgery, including the formation and release of histamine and other toxic substances^16,17^. Thus, in addition to esketamine, azasetron and dexamethasone should also be considered for patients undergoing spinal surgery. Our results also demonstrate that drinking is a risk factor for postoperative opioid self-administration. Pain and alcohol share overlapping neural circuitry, and persistent drinking results in vitamin B1 deficiency, disrupted carbohydrate metabolism, and increased oxidative stress and mitochondrial load^18,19^. These pathological changes may contribute to enhanced cytokine production, increased mitogen-activated protein kinase, and microglial activation, which contribute to the development and maintenance of chronic pain^20^. Finally, our results showed that patients who underwent fixation surgery used more PCA. Fixation surgery causes more tissue and bone damage, resulting in more severe pain. Thus, fixation surgery and drinking are risk factors for more frequent postoperative opioid self-administration.

Previous studies demonstrated sex-based differences in pharmacokinetics and pharmacodynamics^21,22^. Pharmacokinetics represents the dosage of a drug and changes in drug concentrations in the body over time. Drug bioavailability, distribution, metabolism, and excretion affect pharmacokinetics. Gastric emptying, body fat proportion, plasma volume, hepatic enzymes, and renal function all contribute to sex-based differences in pharmacokinetics^23^. Thus, the same plasma concentration of a drug may result in different clinical outcomes due to sex-based differences in pharmacodynamics. We observed significant differences in the effects of azasetron but not esketamine or other pharmacotherapies on postoperative opioid consumption between males and females. A prospective randomized trial may confirm the pharmacological effects of esketamine on postoperative opioid consumption in different sexes. Our study also showed that the analgesic effects of esketamine, dexamethasone, and azasetron were more robust in patients undergoing fixation surgery compared with patients undergoing non-fixation surgery. Patients undergoing fixation surgery experience higher pain intensity and require higher opioid doses to control the pain. Thus, the analgesic effects were statistically significant in patients with higher pain intensity.

Although our study showed that esketamine is associated with fewer postoperative PCA pushes, our study has several limitations. This was a retrospective study and some possible confounding variables, including surgical techniques^24^ and preoperative anxiety levels^25^, could not be determined. In addition, the adverse effects of esketamine^26^, including delirium, hallucination, and nightmares, were not determined in this study. Thus, our results may overestimate the advantage of esketamine. Lastly, esketamine is used to treat depression^27,28^, and the anti-depressive effects could be masked by anesthesia^29^. However, no patients were diagnosed with depression preoperative in this study. Thus, the anti-depressive effects were not observed in our study. A prospective randomized controlled trial should be performed to provide robust support for the effects of esketamine on postoperative opioid self-administration.

## Conclusions

In this study, it was found that a sub-anesthetic dose of esketamine decreased postoperative opioid self-administration after spinal surgery. Therefore, intraoperative esketamine, dexamethasone, and azasetron should be considered for patients undergoing spinal surgery.

### Supplementary Information

Supplementary Tables.

## Data Availability

The data set analyzed during the current study are available from the corresponding author upon reasonable request.

## Abbreviations

ASA: American Society of Anesthesiologists
CRP: C-reactive protein
DM: Diabetes mellitus
IRRs: Incidence rate ratios
IQR: Interquartile range
NRS: Numerical rating scale of pain
NSAID: Nonsteroidal antiinflammatory drug
PCA: Patient-controlled analgesia
SD: Standard deviation
WBC: White blood cell count
