# Supplementary material for: Sub-anesthetic dose of esketamine decreases postoperative opioid self-administration after spine surgery: a retrospective cohort analysis
Source: Sci Rep. 2024 Feb 16;14:3909. doi: 10.1038/s41598-024-54617-5 (PMC10873399; doi:10.1038/s41598-024-54617-5)
Supplement: Supplementary file 1 — Supplementary Tables. [file 41598_2024_54617_MOESM1_ESM.docx]

Supplemental Table 1. Dose of esketamine and postoperative PCA pushes

|  | 0.2 mg/kg n = 28 | 0.3 mg/kg n = 35 | 0.4 mg/kg n = 35 | 0.5 mg/kg  n = 23 | *P* overall | *P* for trend |
| --- | --- | --- | --- | --- | --- | --- |
| PCA pushes | 2.50 [1.00; 5.00] | 2.00 [0.50; 3.00] | 1.00 [0.00; 2.50] | 1.00 [0.00; 3.00] | 0.035 | 0.013 |

Patient-controlled analgesia, PCA

Supplemental Table 2. Sex-specific effects of esketamine and other therapies on postoperative PCA pushes

|  | Sex | Male |  |  | Female |  |  |
| --- | --- | --- | --- | --- | --- | --- | --- |
| Therapies |  | No | yes | P | No | yes | P |
| Esketamine | n | 209 | 55 |  | 260 | 66 |  |
|  | Median (quartile) | 3.00 [1.00; 7.00] | 1.00 [0.00; 4.00] | 0.001 | 2.00 [1.00; 6.00] | 2.00 [1.00; 3.00] | 0.036 |
| Azasetron | n | 54 | 210 |  | 74 | 252 |  |
|  | Median (quartile) | 4.50 [1.00; 8.75] | 2.00 [1.00; 6.00] | 0.063 | 4.00 [2.00; 8.75] | 2.00 [1.00; 4.00] | 0.001 |
| Dexamethasone | n | 81 | 183 |  | 87 | 237 |  |
|  | Median (quartile) | 4.00 [1.00; 9.00] | 2.00 [1.00; 5.00] | 0.038 | 4.00 [1.50; 7.50] | 2.00 [1.00; 4.00] | 0.001 |
| Flurbiprofen | n | 68 | 196 |  | 81 | 245 |  |
|  | Median (quartile) | 2.00 [1.00; 5.25] | 2.50 [1.00; 7.00] | 0.885 | 3.00 [1.00; 6.00] | 2.00 [1.00; 5.00] | 0.305 |
| Dezocine | n | 193 | 71 |  | 239 | 87 |  |
|  | Median (quartile) | 2.00 [1.00; 7.00] | 3.00 [0.50; 7.00] | 0.727 | 2.00 [1.00; 5.00] | 2.00 [1.00; 5.00] | 0.462 |
| Hydromorphone | n | 164 | 100 |  | 229 | 97 |  |
|  | Median (quartile) | 2.50 [1.00; 7.00] | 2.00 [1.00; 6.00] | 0.798 | 2.00 [1.00; 5.00] | 2.00 [1.00; 5.00] | 0.304 |
| Dexmedetomidine | n | 215 | 49 |  | 246 | 80 |  |
|  | Median (quartile) | 3.00 [1.00; 7.00] | 2.00 [1.00; 4.00] | 0.673 | 2.00 [1.00; 5.75] | 2.00 [1.00; 4.00] | 0.308 |

Supplemental Table 3. Surgery type-specific effects of esketamine and other therapies on postoperative PCA pushes

|  | Surgery type | No fixation | |  | Fixation | |  |
| --- | --- | --- | --- | --- | --- | --- | --- |
| Therapies |  | No | yes | P | No | yes | P |
| Esketamine | n | 123 | 20 |  | 346 | 101 |  |
|  | Median (quartile) | 1.00 [0.00; 3.00] | 1.00 [0.00; 2.25] | 0.321 | 3.00 [1.00; 7.75] | 2.00 [1.00; 4.00] | 0.001 |
| Azasetron | n | 26 | 117 |  | 102 | 345 |  |
|  | Median (quartile) | 0.50 [0.00; 2.00] | 1.00 [0.00; 3.00] | 0.141 | 5.50 [2.00; 10.0] | 2.00 [1.00; 6.00] | 0.001 |
| Dexamethasone | n | 38 | 105 |  | 130 | 317 |  |
|  | Median (quartile) | 1.00 [0.00; 2.75] | 1.00 [0.00; 3.00] | 0.336 | 6.00 [2.00; 10.0] | 2.00 [1.00; 5.00] | 0.001 |
| Flurbiprofen | n | 29 | 114 |  | 120 | 327 |  |
|  | Median (quartile) | 1.00 [0.00; 4.00] | 1.00 [0.00; 3.00] | 0.827 | 3.00 [1.00; 6.00] | 3.00 [1.00; 7.00] | 0.721 |
| Dezocine | n | 103 | 40 |  | 329 | 118 |  |
|  | Median (quartile) | 1.00 [0.00; 3.00] | 2.00 [0.00; 4.00] | 0.465 | 3.00 [1.00; 7.00] | 2.00 [1.00; 7.00] | 0.231 |
| Hydromorphone | n | 98 | 45 |  | 295 | 152 |  |
|  | Median (quartile) | 1.00 [0.00; 3.00] | 1.00 [0.00; 3.00] | 0.830 | 3.00 [1.00; 7.00] | 3.00 [1.00; 6.25] | 0.677 |
| Dexmedetomidine | n | 106 | 37 |  | 355 | 92 |  |
|  | Median (quartile) | 1.00 [0.00; 3.00] | 1.00 [0.00; 3.00] | 0.789 | 3.00 [1.00; 7.00] | 2.00 [1.00; 5.00] | 0.433 |
